# Symmetry-adjusted cryo-EM analysis unveils the detailed linker protein CsoS2 interactions within the α-carboxysome shell

**DOI:** 10.1093/plphys/kiaf165

**Published:** 2025-05-09

**Authors:** Jianxun Li, Tianpei Li, Saimeng Wang, Yu-Zhong Zhang, Lu-Ning Liu, Peng Wang

**Affiliations:** Marine Biotechnology Research Center, State Key Laboratory of Microbial Technology, Shandong University, Qingdao 266237, China; Institute of Systems, Molecular and Integrative Biology, University of Liverpool, Liverpool L69 7ZB, UK; Marine Biotechnology Research Center, State Key Laboratory of Microbial Technology, Shandong University, Qingdao 266237, China; Marine Biotechnology Research Center, State Key Laboratory of Microbial Technology, Shandong University, Qingdao 266237, China; MOE Key Laboratory of Evolution and Marine Biodiversity, Frontiers Science Center for Deep Ocean Multispheres and Earth System & College of Marine Life Sciences, Ocean University of China, Qingdao 266003, China; MOE Key Laboratory of Evolution and Marine Biodiversity, Frontiers Science Center for Deep Ocean Multispheres and Earth System & College of Marine Life Sciences, Ocean University of China, Qingdao 266003, China; Institute of Systems, Molecular and Integrative Biology, University of Liverpool, Liverpool L69 7ZB, UK; MOE Key Laboratory of Evolution and Marine Biodiversity, Frontiers Science Center for Deep Ocean Multispheres and Earth System & College of Marine Life Sciences, Ocean University of China, Qingdao 266003, China

## Abstract

Excessive symmetry in cryo-EM data processing can distort key structural details of bacterial microcompartments, highlighting the importance of balanced symmetry for accurate structural insights.

Dear Editor,

Carboxysomes are specialized microcompartments found in all cyanobacteria and certain chemoautotrophic proteobacteria, playing a crucial role in CO_2_ fixation and serving as a central component of bacterial CO_2_-concentrating mechanisms (Kerfeld et al. 2018; Liu 2022). These organelles encapsulate 2 cargo enzymes: Ribulose-1,5-bisphosphate carboxylase/oxygenase (Rubisco) and carbonic anhydrase, using a polyhedral proteinaceous shell. The selective permeability of the carboxysome shell allows bicarbonate (HCO_3_^−^) to enter the carboxysome, where it is converted to CO_2_ by carbonic anhydrase, while restricting the entry of O_2_ (Faulkner et al. 2020; Huang et al. 2022). These natural characteristics promote increased carboxylation activity of Rubisco and reduced photorespiration, substantially improving the competitiveness and survival of microorganisms and contributing substantially to the global carbon cycle.

Carboxysomes can be classified into 2 lineages: α-carboxysomes and β-carboxysomes, which vary in the forms of encapsulated Rubisco, protein composition, and assembly mechanisms. Self-assembly of the α-carboxysome is mediated by the intrinsically disordered protein CsoS2 (Cai et al. 2015; Oltrogge et al. 2020; Ni et al. 2023; Li et al. 2024). CsoS2 functions as a linker protein, connecting cargo enzymes like Rubisco with the shell proteins. It is composed of 3 domains: the N-terminal domain that interacts with Rubisco (Blikstad et al. 2023), the C-terminal domain that anchors to the inner surface of the carboxysome shell (Ni et al. 2023), and the middle region for regulating carboxysome size and shape (Li et al. 2024; Oltrogge et al. 2024). Understanding how CsoS2 mediates shell assembly, cargo encapsulation, and the overall architecture of the α-carboxysome is essential for uncovering the fundamental mechanisms of carboxysome biogenesis and reprogramming carboxysome-based structures for biotechnological applications.

Recent studies using cryo-electron microscopy (cryo-EM) have characterized the complex architecture and assembly mechanisms of α-carboxysome shells. For instance, heterologous expression of minimal shell protein components derived from *Halothiobacillus neapolitanus*, including the hexameric shell protein CsoS1A, pentameric vertex CsoS4A, and CsoS2, allowed for the construction of a *T* = 9 icosahedral α-carboxysome shell with a maximum diameter of 36.9 nm (Ni et al. 2023). Cryo-EM analysis revealed that the C-terminal domain of CsoS2 interacts with multiple neighboring shell proteins via highly conserved, repetitive [IV]TG motifs, which are essential for the assembly of the α-carboxysome shell. The following studies by Zhou et al. (2024) determined the vertex structure of the *T* = 49 shell (86 nm in diameter) from native α-carboxysomes of *Prochlorococcus marinus* under C5 symmetry with a relatively lower resolution (4.2 Å). They found that in addition to the C-terminal domain, the middle region of CsoS2 attaches to the inner surface of the shell also via the conserved [IVL]TG motifs, and 2 different binding patterns of CsoS2 were observed. In our recent study, we generated recombinant α-carboxysome shells containing all shell proteins and the C-terminal domain of CsoS2 (Wang et al. 2024). Under icosahedral (I) symmetry, we obtained cryo-EM structures of a series of α-carboxysome shells (*T* = 9, *T* = 13, *T* = 16, *T* = 19) with a maximum diameter of 54 nm. At near-atomic resolution, we were able to decipher in detail the intricate interactions of CsoS2 with shell proteins, which drive the assembly of α-carboxysome shells. Moreover, our findings showed that CsoS2 is present in 2 distinct conformations within the shell.

In theory, adding symmetry during data processing can improve resolution and provide an effective means for studying the molecular mechanism of CsoS2. However, excessive symmetry can cause over-averaging in specific regions, resulting in the loss of structural information (Zhou et al. 2024). This impedes the detailed understanding of CsoS2 interaction and function. Notably, this issue is not exclusive to vertex maps generated by specific symmetry applications, since maps processed with I symmetry also face similar challenges.

To address this issue, we reanalyzed our recently reported cryo-EM density maps (Wang et al. 2024) without applying any symmetry (C1 symmetry, Supplementary Table S1). Fortunately, we were still able to obtain maps for the *T* = 9 and *T* = 16 α-carboxysome shells at resolutions of 2.77 Å and 3.71 Å, respectively. This allowed us to technically validate the effect of symmetry on the structural information of CsoS2 and offered a detailed mechanistic understanding of its interaction and function within the shell.

The construction of structures using C1 symmetry maps yielded *T* = 9 shells with a diameter of 36.9 nm and *T* = 16 shells with a diameter of 49.2 nm (Fig. 1), which are virtually identical to the structures obtained under I symmetry. Intriguingly, we observed a complete and continuous density at the centroid of the inner surface along the 3-fold axis within the *T* = 16 shell C1 symmetry map, whereas the corresponding position in the I symmetry map showed only limited weaker, fragmented densities (Fig. 1A). Specifically, this relative position coincides with the junction of 3 copies and the 3-fold axis under I symmetry, suggesting that the density discrepancies at the same position in maps of different symmetries are due to symmetrical treatment. High-symmetry processing involves a high degree of data averaging, which can lead to the fragmentation or even the absence of complete density. Nevertheless, in the *T* = 9 C1 symmetry map, no redundant density was detected, indicating that high-symmetry processing is unlikely to greatly impact the structural building in shells with smaller diameters (Fig. 1B).

**Figure 1.**
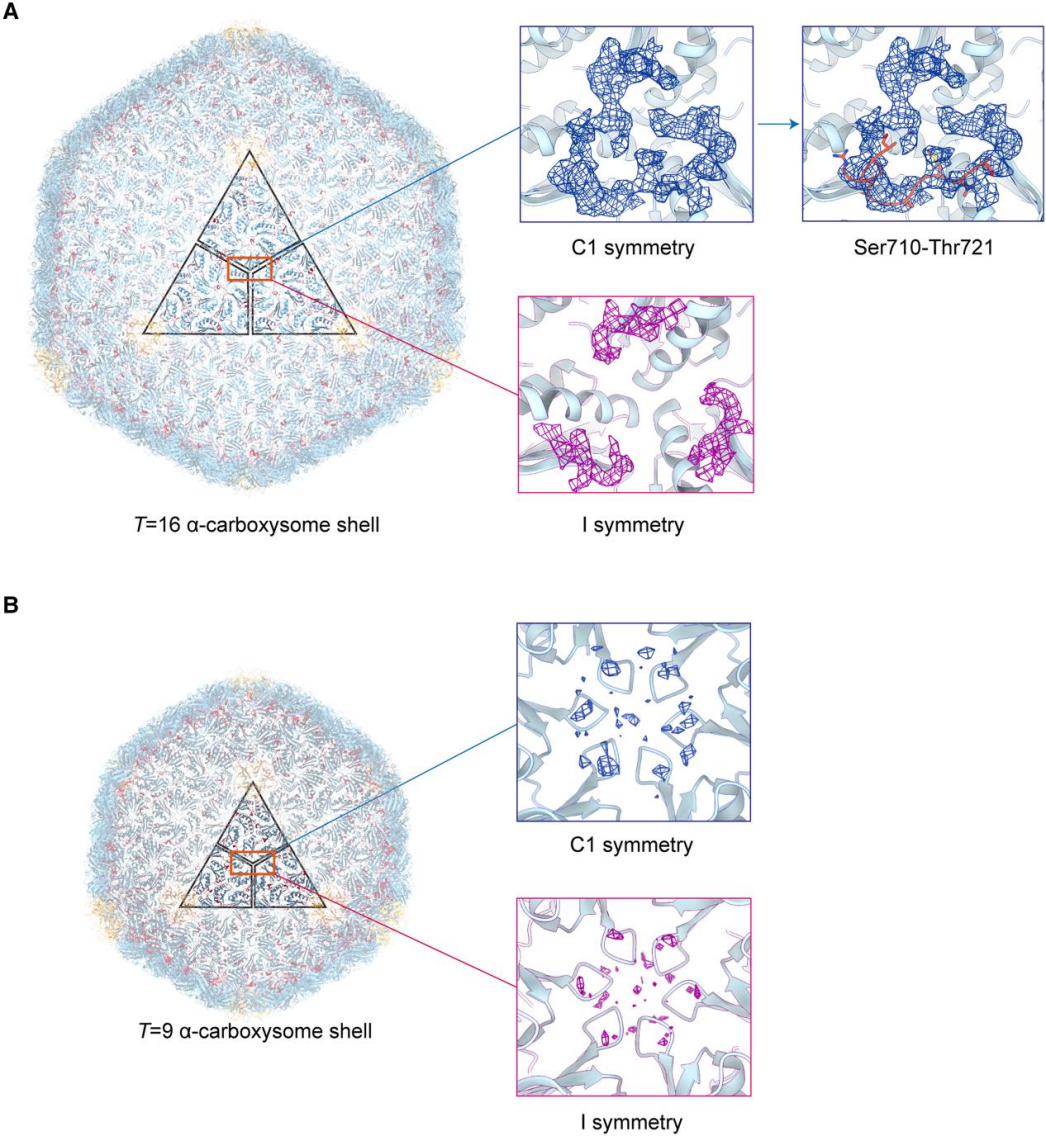
The overall cross-sectional diagram of carboxysome shells and the enlarged density of the suspected CsoS2 at the 3-fold axis in the C1 and I symmetry maps. **A)** The overall structure of *T* = 16 shell (left), the suspected density (middle) and the result of constructing Ser710 to Thr721 of CsoS2 (right). **B)** The overall structure of *T* = 9 shell (left) and the density at the threefold axis of the icosahedron shell (right). The hexamer CsoS1A are colored blue, the pentamer vertexes CsoS4A are colored yellow, and the CsoS2 linker proteins are colored red. The black quadrilateral represents 3 copies on a facet, and the orange square demarcates the relative position of the extra density.

Based on the relative position and conformation of this density on the inner surface of the α-carboxysome shell, we have reason to identify it as the density of CsoS2. Although we were only able to partially build the structure of CsoS2 from Ser710 to Thr721 owing to distortions in the density caused by superposition and averaging of the particles during cryo-EM data processing, this result is a meaningful complement to unveiling the distribution of CsoS2 on the inner surface of the α-carboxysome shell (Fig. 2). From this perspective, we conducted additional analysis on the *T* = 13 and *T* = 19 maps with I symmetry obtained in our recent work (Wang et al. 2024). We also observed faint densities at analogous positions along the asymmetric unit boundaries, although symmetry-induced averaging caused remarkable distortions (Supplementary Fig. S1). Nonetheless, these findings support the notion that the CsoS2 structure is positioned at each interface among three CsoS1A hexamers.

**Figure 2.**
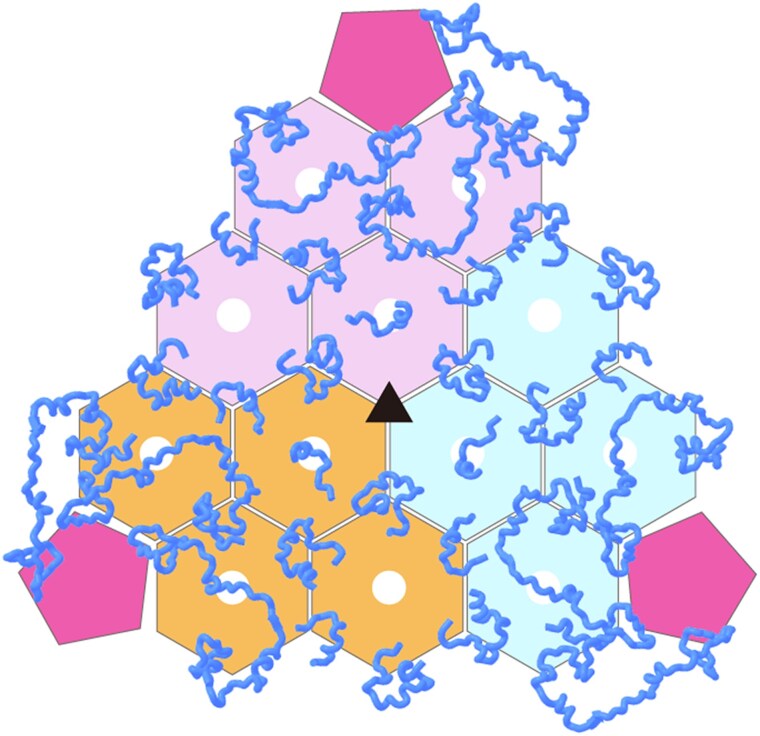
The distribution of the constructed structures and potential positions of CsoS2 binding on the inner surface of the *T* = 16 carboxysome shell. The shell protein modules are simplified to regular polygons, with pentamer located at the shell vertices depicted as purple pentagons, and hexamers belonging to different asymmetric units represented by pink, orange, and light blue hexagons, respectively. The CsoS2 structures constructed on the inner surface of the shell are colored blue, and the extra CsoS2-binding site found in this study is indicated by black triangle.

Our previous results suggest that the binding pattern of CsoS2 to the inner surface of the shell with different sizes is consistent (Supplementary Fig. S2;  Wang et al. 2024). When the shell size exceeds 44.5 nm (*T* = 13), the number of CsoS2 molecules in an asymmetric unit increases to 2, and conformational changes are observed in F3 and F4. The results of this study indicate that there are also potential CsoS2-binding sites at the threefold axis of the facet of larger shells (with a diameter >44.5 nm), thereby augmenting the theoretical number of CsoS2 molecules within a single shell.

In summary, we show that excessive averaging in the highly symmetrical processing of α-carboxysome shell cryo-EM data can distort local densities, potentially leading to the loss of key structural details. Furthermore, the comparison of maps under C1 and I symmetry reveals additional CsoS2-binding sites on the inner surface of the α-carboxysome shell. This study provides valuable insights into the molecular interactions between CsoS2 and the inner surface of the shell. Additionally, our findings provide guidelines for cryo-EM data processing, applicable not only to icosahedral bacterial microcompartment shells such as carboxysome and metabolosomes, but also in other icosahedral structures such as viruses and artificially designed cargo-encapsulating cages that contain scaffolding proteins similar to CsoS2, highlighting the importance of sensible selection between high symmetry for higher-resolution maps and low symmetry for complete and unrefined structural information.

## Supplementary Material

kiaf165_Supplementary_Data

## Data Availability

The details of Cryo-EM data processing for midi-shells are available in Supplementary Fig. S2 of our recently published paper (Wang et al. 2024; https://doi.org/10.1126/sciadv.adr4227). The C1 symmetry cryo-EM density maps for *T* = 16 and *T* = 9 midi-shell in this study have been deposited in the Electron Microscopy Data Bank (EMDB, www.ebi.ac.uk/pdbe/emdb/) with the accession codes EMD-62529 and EMD-62530. The atomic coordinates for *T* = 16 and *T* = 9 midi-shell under C1 symmetry have been deposited in the Protein Data Bank (PDB, www.rcsb.org) with the accession codes 9LY9 and 9LY8. The original I symmetry cryo-EM density maps for *T* = 9, *T* = 13, *T* = 16, and *T* = 19 midi-shell have been deposited in EMDB with the accession codes EMDB-39598, EMD-39601, EMD-39597, and EMD-39596, respectively (Wang et al. 2024).

## Supplementary data

The following materials are available in the online version of this article.

**Supplementary Figure S1**. The density at the binding vacant positions of CsoS2 under I symmetry.

**Supplementary Figure S2**. Comparison of the binding pattern of CsoS2 on the inner surface of carboxysome shells with different sizes.

**Supplementary Table S1**. Cryo-EM data collection, refinement and validation statistics.
