# Supplementary material for: Symmetry-adjusted cryo-EM analysis unveils the detailed linker protein CsoS2 interactions within the α-carboxysome shell
Source: Plant Physiol. 2025 May 9;198(1):kiaf165. doi: 10.1093/plphys/kiaf165 (PMC12062957; doi:10.1093/plphys/kiaf165)
Supplement: kiaf165_Supplementary_Data [file kiaf165_supplementary_data.pdf]

Supplementary Data

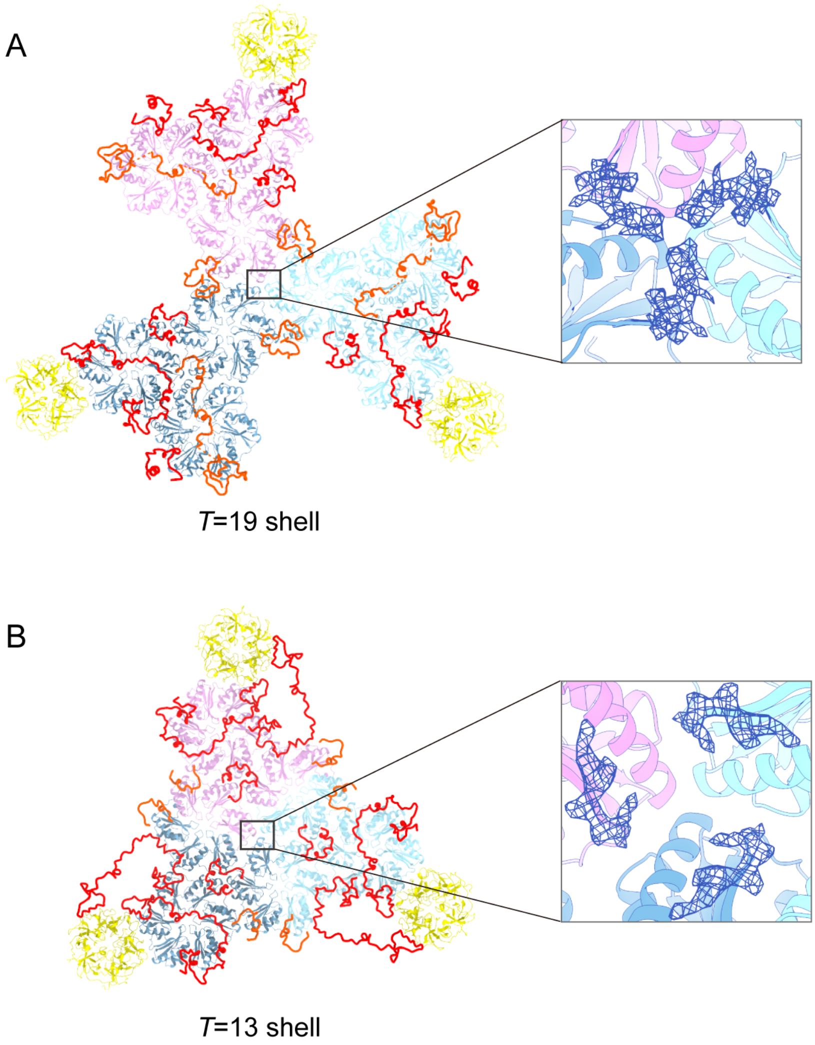

Supplementary Figure S1. The density at the binding vacant positions of CsoS2 under I symmetry. (A) Density at the binding vacant positions of CsoS2 in *T*=19 shell. (B) Density at the binding vacant positions of CsoS2 in *T*=13 shell. The pentamers at vertices are colored yellow, hexamers belonging to different asymmetric units are represented in pink, blue, and light blue, respectively, and CsoS2 in different conformations are depicted in red and orange. The vacant binding sites for CsoS2 are outlined in black, and the local density of their positions in the I symmetry map are enlarged on the right.

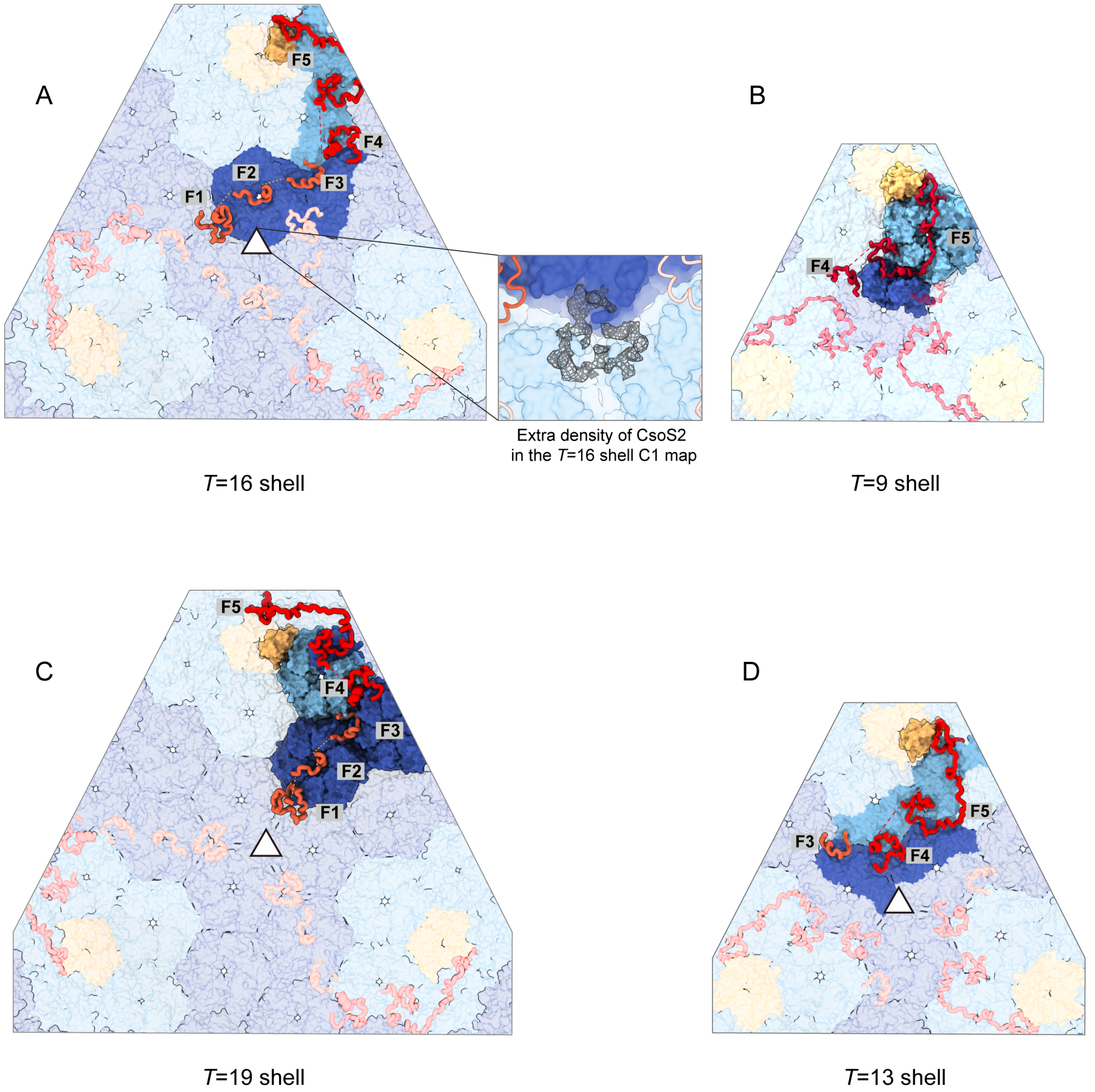

Supplementary Figure S2. Comparison of the binding pattern of CsoS2 on the inner surface in carboxysome shells with different sizes. (A) *T*=16 shell, the extra density of CsoS2 in the *T*=16 shell C1 symmetry map is enlarged. (B) *T*=9 shell. (C) *T*=19 shell. (D) *T*=13 shell. The pentamers are colored yellow. Hexamers at different relative positions are represented in different colors: light blue, shell hexamers adjacent to pentamers; blue, hexamers that are not adjacent to pentamers. Fragments (labeled) belonging to two different CsoS2 chains are colored red and orange, respectively. Only CsoS2 from three of the asymmetric units are shown on each facet for clarity. One of the asymmetric units on facet is highlighted by reducing transparency. The white triangles represent the extra binding sites of CsoS2 identified in this study.

Supplementary Table S1. Cryo-EM data collection, refinement and validation statistics.

Data collection and processing	*T*=9 (C1)	*T*=16 (C1)
Magnification (nominal)	81,000	81,000
Voltage (kV)	300	300
Detector	Gatan K3	Gatan K3
Electron exposure (e^-/Å^2)	50.0	50.0
Defocus range (μm)	-1.2~-2.5	-1.2~-2.5
Pixel size (Å)	0.53	0.53
Symmetry imposed	C1	C1
Initial particle images (no.)	92,101	92,101
Final particle images (no.)	40,177	12,775
FSC threshold	0.143	0.143
Map resolution (Å)	2.77	3.71
Refinement		
Initial model used (PDB code)	8B12	8B12
Model resolution (Å)	1.86	1.86
FSC threshold	0.143	0.143
Model composition		
Non-hydrogen atoms	403140	716166
Protein residues	56100	99978
*B*factor (Å^2)		
Protein	19.20	37.58
R.m.s deviations		
Bond lengths (Å)	0.022	0.016
Bond angles (°)	2.116	2.347
Validation		
MolProbity score	1.85	1.93
Clashscore	8.17	8.77
Poor rotamers (%)	3.45	4.02
Ramanchandran plot		
Favored (%)	99.36	98.55
Allowed (%)	0.64	1.44
Disallowed (%)	0	0.01
Accession codes	EMD-62530 PDB 9LY8	EMD-62529 PDB 9LY9
